# Are Sick Individuals Weak Competitors? Competitive Ability of Snails Parasitized by a Gigantism-Inducing Trematode

**DOI:** 10.1371/journal.pone.0079366

**Published:** 2013-10-31

**Authors:** Otto Seppälä, Anssi Karvonen, Marja Kuosa, Maarit Haataja, Jukka Jokela

**Affiliations:** 1 Institute of Integrative Biology, ETH Zürich, Zürich, Switzerland; 2 Department of Aquatic Ecology, Eawag, Dübendorf, Switzerland; 3 Department of Biological and Environmental Science, University of Jyväskylä, Jyväskylä, Finland; 4 Department of Biology, University of Oulu, Oulu, Finland; 5 Research Funding Services, University of Helsinki, Helsinki, Finland; Technion-Israel Institute of Technology Haifa 32000 Israel, Israel

## Abstract

Parasitized individuals are often expected to be poor competitors because they are weakened by infections. Many trematode species, however, although extensively exploiting their mollusc hosts, also induce gigantism (increased host size) by diverting host resources towards growth instead of reproduction. In such systems, alternatively to reduced competitive ability due to negative effects of parasitism on host performance, larger size could allow more efficient resource acquisition and thus increase the relative competitive ability of host individuals. We addressed this hypothesis by testing the effect of a trematode parasite *Diplostomum pseudospathaceum* on the competitive ability of its snail host *Lymnaea stagnalis*. We experimentally examined the growth of snails kept in pairs in relation to their infection status and intensity of resource competition (i.e. food availability). We found that parasitized snails grew faster and their reproduction was reduced compared to unparasitized individuals indicating parasite-induced gigantism. However, growth of the snails was faster when competing with parasitized individuals compared to unparasitized snails indicating reduced competitive ability due to parasitism. The latter effect, however, was relatively weak suggesting that the effects of the parasite on snail physiology may partly override each other in determining competitive ability.

## Introduction

Parasites often have profound effects on host physiology, behavior and survival [1], [2]. These effects could modify competitive interactions within host populations, which may have wide ecological and evolutionary consequences by altering host and parasite population dynamics [3]–[5] as well as parasite virulence (i.e. disease severity) [6]. Parasites that reduce host population density by inducing mortality can be especially important in relaxing competitive interactions among hosts, and thus increasing the availability of resources for unparasitized individuals (i.e. competitive release) [7]. For instance, emergence of adult treehole mosquitoes *Aedes sierrensis* is improved when larval populations are parasitized by a ciliate *Lambornella clarki* under limited, but not under unlimited, food conditions [4].

The effects of parasites with weaker influence on host population density are, however, often nontrivial to predict. In such cases, the effect of parasitism on competitive ability of host individuals is necessary to be quantified. To date, such studies are scarce, and their results are somewhat conflicting. Earlier studies from different host–parasite interactions have reported reduced [6], [8]–[11], neutral [12], [13] and even increased [11], [14], [15] competitive ability of the parasitized hosts. This could be because the nature of each particular host–parasite interaction may determine parasite's effect on competitive interactions among hosts. For example, the way how parasites utilize host resources, how they affect host physiology and behavior, and how hosts respond to infections (e.g. immune defense) may lead to the observed variation across study systems. For instance, intracellular bacteria *Caedibacter* spp. of freshwater ciliates *Paramecium* spp. increase the competitive ability of parasitized hosts although they negatively influence other aspects of host performance [15]. This effect is due to an advantage in interference competition as parasitized paramecia release a toxic form of the bacterium that kills unparasitized individuals [15]. Because of the potential importance of such system specific effects, more studies from host–parasite interactions with different characteristics are needed to understand the role of parasites in determining host competitive ability. In this study, we examined the effect of a castrating trematode on the competitive ability of its snail host.

In mollusc–trematode interactions, exploitation of host resources by parasites is often very extensive as trematodes use a large proportion of host resources for their own growth and reproduction [16]. Thus, hosts are often weakened by infections, which leads, for example, to their increased mortality [17]–[19]. Interestingly, trematodes do not only utilize host resources but they can also modify resource allocation among host traits. Trematodes occupy the gonad tissue of snails and eventually castrate them. This releases resources from reproduction to somatic growth, which sometimes leads to increased size of parasitized individuals known as gigantism [20]–[22]. When parasites modify not only the amount of host resources but also their allocation among different traits, the overall effect of parasitism on host competitive ability is difficult to predict. This is because (1) competitive ability of parasitized hosts may be reduced if they are in poor physiological condition due to host exploitation by the parasite or (2) potential gigantism may give them a competitive advantage as large individuals are often more efficient in resource acquisition and stronger in interference competition [23]. Here, we experimentally investigated the effect of a parasite *Diplostomum pseudospathaceum* (Trematoda) on the competitive ability of its snail host *Lymnaea stagnalis*. We contrasted the above hypotheses by experimentally examining the competitive ability of snails in relation to their infection status and the intensity of resource competition (i.e. food availability).

## Materials and Methods

### Ethics Statement

This study was carried out in accordance with the laws governing animal experimentation in Finland and Switzerland. Use of gulls and the methods used for their maintenance in the laboratory were approved by the Lab-Animal Care and Use Committee of the University of Jyväskylä and the State Provincial Office of Western Finland. In Finland and Switzerland, work with snails does not require permissions. The study did not involve endangered or protected species. No specific permits were required for the field operations as the used water bodies are not private property or nature reserves.

### Study System

*Lymnaea stagnalis* is a hermaphroditic freshwater snail inhabiting shallow littoral zones of stagnant waters such as lakes and ponds in Holarctic region. It is an important host for a community of parasites including several trematode species [24]–[26].

*Diplostomum pseudospathaceum* is one of the most common trematode parasites infecting *L. stagnalis* [24], [25]. It has a three-host life cycle with a bird definitive host, and snail and fish intermediate hosts. The parasite matures in the intestine of fish-eating birds, where it reproduces sexually. Eggs of the parasite are released to water with the birds' feces and hatch into free-swimming miracidia larvae. Miracidia infect snails, where they penetrate into snail gonads and develop into sporocysts. Sporocysts multiply asexually and take over the gonad tissue. The development of patent infection takes several weeks depending on the water temperature. During this period, parasite castrates the snail. Sporocysts produce free-swimming cercaria larvae through asexual reproduction, and an individual snail can produce thousands of cercariae per day for several weeks [19], [27]. This extensive host exploitation leads to increased mortality of parasitized snails [19]. Cercariae of the parasite infect fish by penetrating the gills and skin after which they migrate to the eye lenses where they develop into metacercariae. For successful completion of the life cycle, parasitized fish has to be eaten by a piscivorous bird.

### Experimental Animals

Snails for this study came from a laboratory stock population originating from a pond in Kleinandelfingen in Switzerland (47°36′N, 8°40′E). The population was maintained in the lab for five years (roughly 2–3 generations per year). In mid May 2006, we collected 400 egg clutches from the stock population and placed them in two 200 L tanks (200 egg clutches per tank) with aged tap water and biological filtration. After hatching, we fed the snails with fresh lettuce *ad libitum*, and supplied the tanks with chalk to provide calcium for the development of snails' shells.

We produced parasite eggs to infect snails (see below) under laboratory conditions using two herring gull (*Larus argentatus*) chicks. In the end of May 2006, we collected gull eggs that were close to hatching from nests at Lake Konnevesi in Finland (62°37′N, 26°21′E). We brought the eggs to the laboratory and placed them in an incubator to hatch. We fed the chicks with previously frozen fish to ensure that they did not get any parasites. Two weeks after hatching, we exposed the chicks to *Diplostomum* infection by feeding them with lenses of several parasitized roach (*Rutilus rutilus*) individuals captured from Lake Konnevesi. We gave lenses with a total of 200–300 *Diplostomum* metacercariae to each chick within small pieces of fish. The exposure corresponded to natural parasite abundances (i.e. number of parasites in a host) [28] observed in gulls [29]. It is important to note that the used roach may have been parasitized by several different *Diplostomum* species [30]. However, *L. stagnalis* snails are only susceptible to *D. pseudospathaceum* in Finland [31]. Few days after the infection, we collected about 60000 parasite eggs from gull feces using a 50 µm mesh sieve. We stored the eggs on Petri dishes in small amounts of water until the exposure of snails. We subsequently euthanized the gulls using carbon dioxide.

We started the parasite exposures to produce experimental snails when the snails were nine weeks old. We placed the snails in 20 L boxes (23 boxes; 70 snails per box) with aged tap water and fed them with fresh lettuce. In 13 randomly selected boxes, we exposed the snails to infection by placing a cup with 2800 parasite eggs in each box. We covered the cups with nets to prevent the snails from eating the eggs but allowing hatched miracidia larvae to exit the cups and infect the snails. At the time of starting the exposures, parasite eggs were close to hatching based on their developmental stage that we checked visually. We maintained the exposed snails under these conditions for 26 days and carefully changed half of the water in each box once a week. In 10 boxes, we kept the snails unexposed and treated them as described above except for the parasite exposure. We exposed more snails to infection than what we left unexposed to compensate expected mortality of parasitized snails [19]. After the exposures, we marked the snails by making a dot on their shell using nail polish (we used different colors for exposed and unexposed individuals).

### Experimental Design

We started the competition experiment in early August 2006 when the experimental snails were 9.8–33.7 mm long. Already at that time (26 days after the beginning of the parasite exposures) parasitized snails were larger than unparasitized snails [analysis of variance (ANOVA): *F*_2,431_ = 10.516, *p*<0.001; pairwise contrasts: *p*<0.001 for both; see infection categories below] indicating gigantism.

In the experiment, we randomly assigned experimental snails into the following pairs: (1) unexposed/unexposed, (2) unexposed/exposed, (3) exposed/unexposed, and (4) exposed/exposed (120 pairs per category), where the first term refers to the focal individual (i.e. followed over the experiment; see below) and the second term to its competitor. After that, we randomly assigned 60 pairs from each category into ‘*ad libitum* food supply’ and another 60 pairs into ‘reduced food supply’ treatments to manipulate the intensity of resource competition between snails. In both feeding treatments, we fed the snails with fresh lettuce every second day. In *ad libitum* food supply, we adjusted the amount of lettuce so that the snails were not able to eat all the lettuce that was provided to them between the feeding days. In reduced food supply, we provided each pair with half a portion of the average lettuce consumption of snails of similar size (i.e. consumption of one individual; 1st week: 0.3 g every second day, 2nd week: 0.4 g every second day, 3rd–10th week: 0.5 g every second day). We used earlier information about food consumption of snails from the same stock population (Seppälä O., unpublished data) to determine the amount of lettuce. We maintained the pairs of snails in 0.3 L plastic cups with net bottoms placed into six 400 L tanks with aged tap water and biological filtration (80 pairs per tank), and fed them according to the feeding treatments for ten weeks. We measured the shell length of each focal individual to the nearest 0.1 mm in two-week-intervals to determine their growth.

After the experiment, we removed the competitors and possible egg clutches from the cups. We determined the infection status of each competitor by removing the snail from its shell, and examining the presence of parasite sporocysts under a microscope. After that, we maintained the focal individuals in their original cups and fed them with fresh lettuce *ad libitum* to determine the effect of parasite on snail reproduction. We fed all snails *ad libitum* to maximize their reproduction in order to obtain a reliable estimate of the extent of parasite-induced castration. After 10 days maintenance, we recorded which snails had laid eggs, counted the number of produced eggs, and determined their infection status as described above. Of the snails that we exposed to miracidia, 41.5% became parasitized. Thus, we had three categories for the infection status of the experimental snails: unexposed, exposed but unparasitized, and exposed and parasitized. Therefore, the final categories for pairs of competing snails were (1) unexposed/unexposed, (2) unexposed/exposed but unparasitized, (3) unexposed/exposed and parasitized, (4) exposed but unparasitized/unexposed, (5) exposed but unparasitized/exposed but unparasitized, (6) exposed but unparasitized/exposed and parasitized, (7) exposed and parasitized/unexposed, (8) exposed and parasitized/exposed but unparasitized, and (9) exposed and parasitized/exposed and parasitized, where the first term refers to the focal individual and the second term to its competitor. A total of 39 snails died during the experiment and we were unable to determine the infection status from seven individuals. Since the mortality of snails during the experiment was generally low (8.1%), we could not use survival as an additional variable to examine the effects of our experimental treatments. Therefore, we excluded these individuals from the data.

### Statistical Analyses

We used specific growth rate [32] of the snails during the competition experiment as a measure of their performance. Snails' growth during the experiment followed a typical power function (Figure S1), for which the specific growth rate (ln*S*_2_ - ln*S*_1_)/Δ*t* is a linear function of logarithm of size (ln*S*) [where *S*_1_ and *S*_2_ represent size at the beginning and at the end of the time period Δ*t* (10 weeks in our study), respectively, and *S* is their geometric mean] [32]. We then analyzed the variation in the specific growth rate of focal individuals using an analysis of covariance (ANCOVA). We used a model with feeding treatment (*ad libitum* food supply, reduced food supply), infection status of focal individual (unexposed, exposed but unparasitized, exposed and parasitized) and infection status of competitor (unexposed, exposed but unparasitized, exposed and parasitized) as fixed factors, and ln*S* as a covariate. When a statistically significant effect of infection status of focal individual and/or competitor was observed in the above analysis, we conducted pairwise comparisons among different infection categories using specific contrasts.

To estimate the effect of parasite infection on reproduction of snails, we first analyzed whether the proportion of snails (focal individuals) that laid eggs after the competition experiment differed across infection categories. We used a generalized linear model where the reproductive status of snails (laid eggs, did not lay eggs) was used as a response variable (binomial distribution, logit link function), and infection status (unexposed, exposed but unparasitized, exposed and parasitized) and feeding treatment during the competition experiment (*ad libitum* food supply, reduced food supply) as fixed factors. We included feeding treatment during the competition experiment as a factor although all snails were fed *ad libitum* during the test for reproduction (see above). This was because snails' resource levels could be affected by the long-term feeding treatment during the study and we wanted to control its possible effect in the analysis. After that, we examined the effect of infection on egg production in snails that reproduced using a generalized linear model with the number of produced eggs as a response variable (Poisson distribution, log link function) and a similar model as above. When a statistically significant effect of infection status was observed in the above analyses, we conducted pairwise comparisons among different infection categories using specific contrasts. We performed all statistical analyses using IBM SPSS 20.0 (IBM, Armonk, NY, USA) software.

## Results

**Figure 1:**
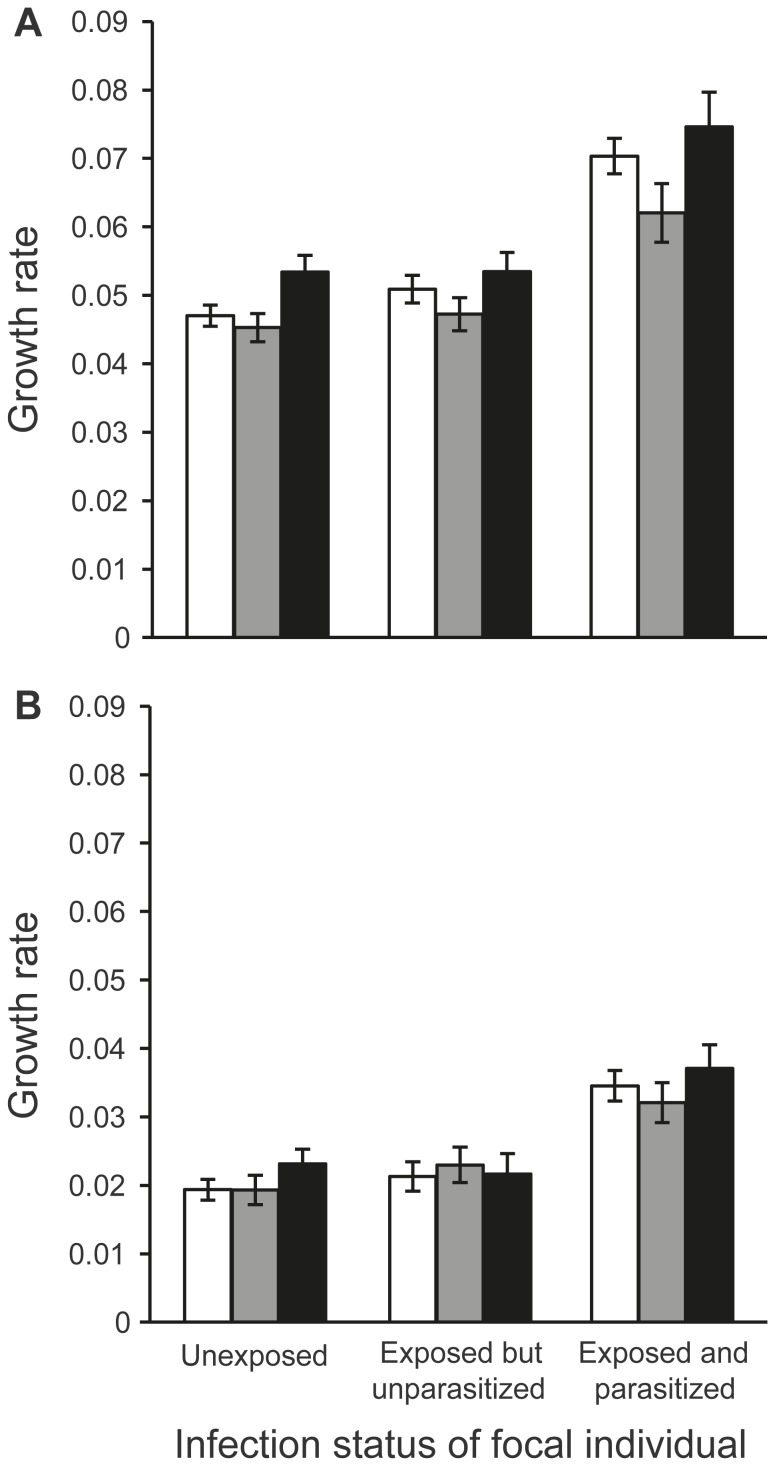
Growth rate of *Lymnaea stagnalis* snails during the experiment. Bars (size-adjusted mean ± SE) show the specific growth rate of focal individuals with different infection status (unexposed, exposed but unparasitized, exposed and parasitized; parasite: *Diplostomum pseudospathaceum*) maintained together with another snail individual [competitor; unexposed (white), exposed but unparasitized (grey), exposed and parasitized (black); parasite: *D. pseudospathaceum*] under (A) *ad libitum* food supply and (B) reduced food supply (i.e. half of the average food consumption) for ten weeks.

Food limitation reduced the growth of snails (focal individuals) during the experiment (Figure 1, Table 1). Thus, we were able to manipulate the amount of external resources for snails using feeding treatments so that resource competition among them should be intensified under reduced food supply compared to *ad libitum* food supply.

**Table 1:** ANCOVA for the specific growth rate of *Lymnaea stagnalis* snails during the experiment.

Source	df	MS	*F*	η^2^ (%)	*p*
Feeding treatment (F)	1	0.063	498.195	32.8	< 0.001
Infection status of focal individual (I_F_)	2	0.006	49.357	6.5	< 0.001
Infection status of competitor (I_C_)	2	0.001	5.210	0.7	0.006
Size	1	0.061	484.366	31.9	< 0.001
F×I_F_	2	0.000	1.985	0.3	0.139
F×I_C_	2	0.000	1.897	0.2	0.151
I_F_×I_C_	4	0.000	0.832	0.2	0.505
F×I_F_×I_C_	4	0.000	0.145	0.0	0.965
Error	415	0.000			

Factors are feeding treatment [*ad libitum* food supply, reduced food supply (i.e. half of the average food consumption)], infection status of focal individual (unexposed, exposed but unparasitized, exposed and parasitized; parasite: *Diplostomum pseudospathaceum*) and infection status of competitor (unexposed, exposed but unparasitized, exposed and parasitized; parasite: *D. pseudospathaceum*). Snail size (ln of geometric mean of initial and final size) was used as a covariate. η^2^ shows the proportion of total variance explained by each factor.

Parasite infection directly affected both growth and reproduction of focal snails. First, parasitized snails had a higher specific growth rate compared to unexposed and exposed but unparasitized individuals (Figure 1, Table 1, pairwise contrasts: *p*<0.001 for both). The growth of unexposed and exposed but unparasitized snails did not differ from each other (pairwise contrast: *p* = 0.202). Second, the proportion of focal individuals that laid eggs after the competition experiment was lower in parasitized snails (estimated marginal mean ± SE = 8.8±3.4%) compared to unexposed (estimated marginal mean ± SE = 83.5±2.5%) and exposed but unparasitized (estimated marginal mean ± SE = 82.6±3.3%) individuals (generalized linear model: Wald χ^2^ = 76.926, df = 2, *p*<0.001, pairwise contrasts: *p*<0.001 for both). Unexposed and exposed but unparasitized snails did not differ from each other (pairwise contrast: *p* = 0.822). Furthermore, from snails that reproduced, parasitized individuals laid fewer eggs (estimated marginal mean ± SE = 30.6±4.1) compared to unexposed (estimated marginal mean ± SE = 112.8±0.8) and exposed but unparasitized (estimated marginal mean ± SE = 114.9±1.2) snails (generalized linear model: Wald χ^2^ = 96.582, df = 2, *p*<0.001, pairwise contrasts: *p*<0.001 for both). Unexposed and exposed but unparasitized snails did not differ from each other in egg-laying (pairwise contrast: *p* = 0.148).

Despite of gigantism, parasite infection reduced competitive ability of snails indicated by the faster growth of focal individuals when kept together with parasitized snails rather than unexposed or exposed but unparasitized individuals (Figure 1, Table 1, pairwise contrasts: *p*≤0.037 for both). Growth of snails did not differ statistically significantly when kept together with unexposed and exposed but unparasitized competitors (pairwise contrast: *p* = 0.088).

## Discussion

Parasites have a potential to modify ecological interactions among free living organisms by altering their physiology and behavior. In the present study, we examined the effect of a trematode parasite *D. pseudospathaceum* on the competitive ability of its snail host *L. stagnalis*. In mollusc–trematode interactions, exploitation of host resources by parasites is often very extensive [16], [27], which weakens the hosts [17]–[19], and could reduce their competitive ability. Many trematodes, however, castrate their hosts and direct host resources towards somatic growth leading to gigantism [20]–[22]. This could increase host competitive ability as large individuals are often more efficient in resource acquisition and stronger in interference competition [23]. By examining the growth of coexisting snails in relation to their infection status, we found that *D. pseudospathaceum* induced gigantism in parasitized snails, but despite of this, reduced their competitive ability. The latter effect, however, was weak.

Our results showing increased size/growth and reduced reproduction in parasitized snails are in line with the idea of parasite-induced gigantism shown in other mollusc–trematode interactions [20]–[22]. In our study, parasitized snails (focal individuals) were larger than unparasitized individuals already at the beginning of the competition experiment (i.e. after 26 days of parasite exposure), and they grew faster during the experiment. Furthermore, the reproductive output of parasitized snails was strongly reduced indicating parasite-induced castration that makes increased growth possible. The effects of infection status on snails' growth and reproduction were only seen between parasitized and unparasitized individuals. Within the group of unparasitized snails, unexposed and exposed but unparasitized individuals did not differ from each other. This suggests no apparent energetic cost of activated immune defense following the exposure to parasites [33], [34] in this system. However, it is possible that snails were able to compensate for increased energetic demands of the immune challenge by increasing food consumption [34].

In addition to the above effects, growth of the snails was faster when competing with parasitized individuals compared to unparasitized snails, which indicates reduced competitive ability due to parasitism. Impaired competitive ability of parasitized hosts has been observed also in some other host–parasite interactions [6], [8]–[11]. For example, the microsporidian parasite *Vavraia culicis* prolongs the larval developmental time of its host *Aedes aegypti*, and has the strongest negative effect when parasitized larvae grow under competition with unparasitized individuals [6]. Similarly, insect parasitoids have been shown to reduce the resource holding potential of their hosts [35]. In our study, however, the effect of parasitism on competitive ability of snails was weak. This was because infection status of competitor explained only 0.7% of the total variance in the growth of focal individuals, which was about one tenth of the proportion of variance explained by the direct effect of parasitism (i.e. infection status of focal individual). Together with earlier findings from other study systems [12], [13], this suggests that all parasites do not necessarily have a strong negative influence on host competitive ability even when they have strong effects on host physiology and performance. For example, in farmed whitefish (*Coregonus lavaretus*), *Diplostomum* eye flukes do not affect the growth of fish in mixed shoals of parasitized and unparasitized individuals despite of the impaired vision of fish caused by parasite-induced cataracts [12].

In our study, the lack of strong negative (owing to host exploitation) or positive (owing to gigantism) effect on host competitive ability may be due to at least two different reasons. First, such effects may partly override each other in determining the competitive ability of snails, leading only to the small negative effect observed in this study. Second, in our study system, the effect of parasitism on competitive ability may depend on the age and developmental stage of infection as the intensity of host exploitation by *D. pseudospathaceum* may vary over the course of infection. In our experiment, competitive ability of snails was examined during the development of sporocysts in snail tissues (i.e. developed parasite cercariae were observed only in few snails when dissected, and their numbers were low). It is possible that energetic costs of infection are higher after sporocysts are fully developed and cercarial production is taking place as an individual snail can produce thousands of cercariae per day [19], [27]. Therefore, also negative effects of infection including reduced competitive ability could become more pronounced at later stages of infection.

Interestingly, the effect of the parasite on competitive ability of snails was consistent between different feeding treatments (*ad libitum* food supply, reduced food supply) in our study. If parasitism affected only resource competition among snails, its effect could be expected to be strongest in the reduced feeding treatment. This is because competition for common resources should be most intense when resources are limited. Thus, our finding suggests that also other mechanisms than modified resource competition may play an important role in this system. For example, direct interactions (e.g. interference) between snails could be important in determining the strength of competition. Reduced locomotion of snails parasitized by trematodes has been found in another snail–trematode interaction [21]. Such an effect could explain our result by modifying direct interactions among snail hosts independently of resource availability. The actual mechanism behind this result, however, remains to be investigated.

To conclude, we found that the trematode *D. pseudospathaceum* reduced competitive ability of its snail host *L. stagnalis*. This effect, however, was relatively weak possibly because potential negative (owing to host exploitation) and positive (owing to gigantism) effects on snails may partly override each other in determining competitive ability. In natural snail populations, the observed effect of the parasite on competition among host individuals may, however, reduce competitive interactions especially in dense populations with high parasite prevalence. Under such conditions, the small effects observed at individual level may add up to significantly increase the availability of resources for unparasitized snails.

## Supporting Information

Figure S1 Change in shell length of *Lymnaea stagnalis* snails during the experiment. Error bars (mean ± SE) show the size of focal individuals with different infection status [unexposed (white), exposed but unparasitized (grey), exposed and parasitized (black); parasite: *Diplostomum pseudospathaceum*] maintained together with another snail individual [competitor; unexposed (□), exposed but unparasitized (◊), exposed and parasitized (○); parasite: *D. pseudospathaceum*] under (A) *ad libitum* food supply and (B) reduced food supply (i.e. half of the average food consumption). The snails were maintained ten weeks, and their size was measured in two-week-intervals.
(TIF)
