# Supplementary figures and images for: Are Sick Individuals Weak Competitors? Competitive Ability of Snails Parasitized by a Gigantism-Inducing Trematode
Source: PLoS One. 2013 Oct 31;8(10):e79366. doi: 10.1371/journal.pone.0079366 (PMC3814966; doi:10.1371/journal.pone.0079366)

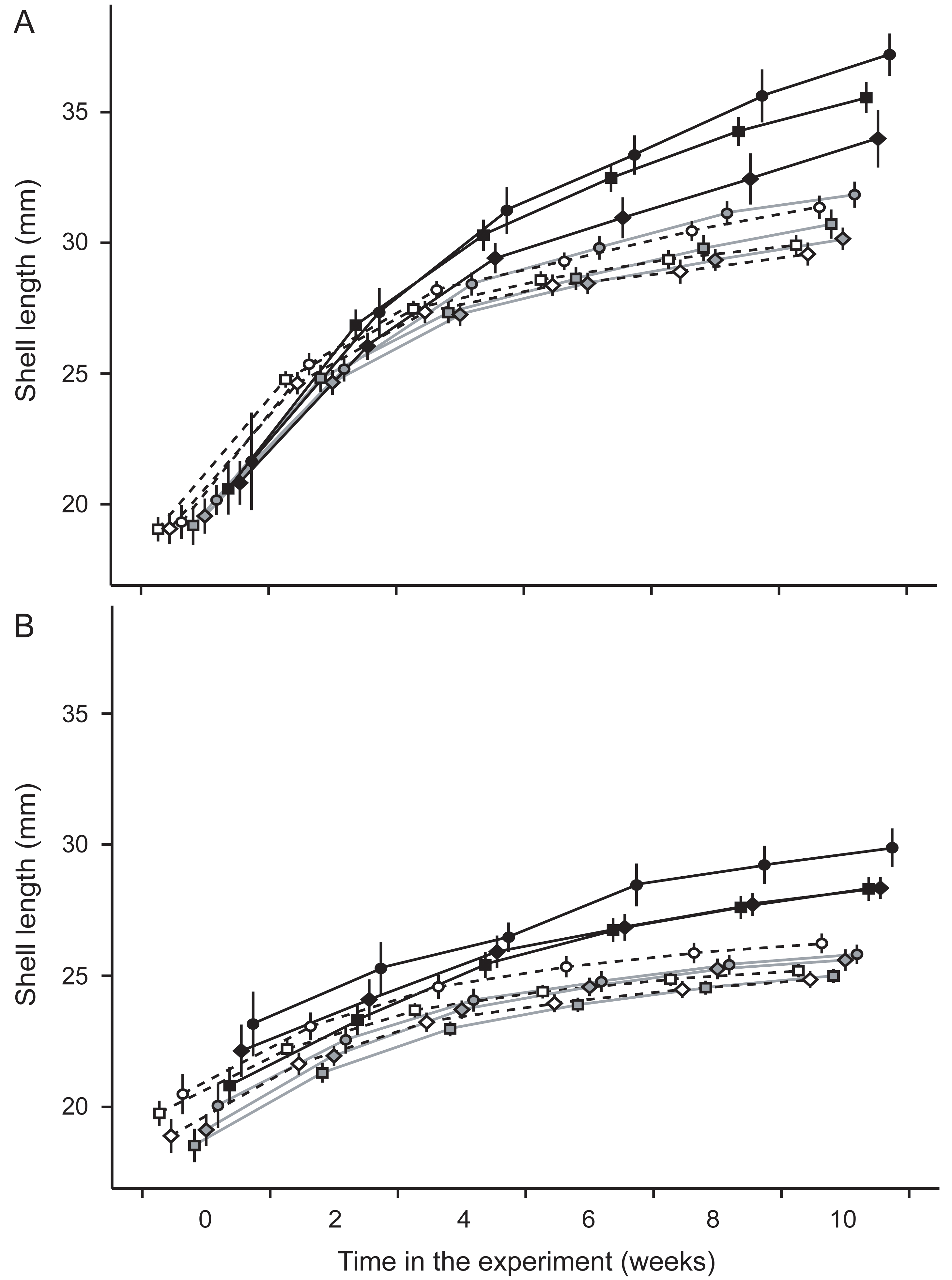

Supplement: Figure S1 — Change in shell length of Lymnaea stagnalis snails during the experiment. Error bars (mean ± SE) show the size of focal individuals with different infection status [unexposed (white), exposed but unparasitized (grey), exposed and parasitized (black); parasite: Diplostomum pseudospathaceum] maintained together with another snail individual [competitor; unexposed (□), exposed but unparasitized (◊), exposed and parasitized (○); parasite: D. pseudospathaceum] under (A) ad libitum food supply and (B) reduced food supply (i.e. half of the average food consumption). The snails were maintained ten weeks, and their size was measured in two-week-intervals. (TIF) [file pone.0079366.s001.tif]
